# Development of a physical map of the soybean pathogen *Fusarium virguliforme *based on synteny with *Fusarium graminearum *genomic DNA

**DOI:** 10.1186/1471-2164-8-262

**Published:** 2007-08-03

**Authors:** Jeffry L Shultz, Sikander Ali, Linda Ballard, David A Lightfoot

**Affiliations:** 1 Currently, USDA-ARS, Crop Genetics and Production Research Unit, PO Box 345, Stoneville, MS, 38776, USA; 2 Institute of Industrial Biotechnology, GC University Lahore, Pakistan; 3 Mississippi Valley State University, Itta Bena, MS. Currently, USDA-ARS MSA Genomics Laboratory, P.O. Box 345, Stoneville, MS, 38776, USA; 4 Dept. of Plant, Soil and Agricultural Systems, Genomics and Biotechnology Core Facility, Center for Excellence in Soybean Research, Southern Illinois University, Carbondale IL, 62901, USA

## Abstract

**Background:**

Reference genome sequences within the major taxa can be used to assist the development of genomic tools for related organisms. A major constraint in the use of these sequenced and annotated genomes is divergent evolution. Divergence of organisms from a common ancestor may have occurred millions of years ago, leading to apparently un-related and un-syntenic genomes when sequence alignment is attempted.

**Results:**

A series of programs were written to prepare 36 Mbp of *Fusarium graminearum *sequence in 19 scaffolds as a reference genome. Exactly 4,152 Bacterial artificial chromosome (BAC) end sequences from 2,178 large-insert *Fusarium virguliforme *clones were tested against this sequence. A total of 94 maps of *F. graminearum *sequence scaffolds, annotated exonic fragments and associated *F. virguliforme *sequences resulted.

**Conclusion:**

Developed here was a technique that allowed the comparison of genomes based on small, 15 bp regions of shared identity. The main power of this method lay in its ability to align *diverged *sequences. This work is unique in that discontinuous sequences were used for the analysis and information not readily apparent, such as match direction, are presented. The 94 maps and JAVA programs are freely available on the Web and by request.

## Background

The soybean pathogen *Fusarium virguliforme *[1-3] (ex *Fusarium solani *f.sp. *glycines*) causes soybean sudden death syndrome (SDS). SDS has caused significant yield losses in soybean since it was first noted in 1980 [4]. *F. virguliforme *is a member of the *Martiella *section, a Fusarium plant disease complex that contains many related pathogens of the worlds major crops [5,6]. *Fusarium virguliforme *is a hemibiotrophic plant pathogen with a wide host range [1,5]. The perfect stage is unknown and all extant strains appear to be of a single mating type [7]. Tools for analysis of this economically important genome have become a high priority, with two BAC libraries and a collection of BAC-end sequences already developed [8-12]. These BAC-end sequences (BES) produce two reads from each discrete clone, and present 1,000–1,600 base pairs of genomic DNA for analysis. A map of ordered, joined sequences can result if sufficient BACs are end-sequenced [13].

A reference genome is defined here as a genome to which phenotypic and/or genotypic traits of other genomes may be compared. The comparison of genotypic traits between both related and distant organisms relies on the information available for the reference genome. This information may be as broad as marker order and karyotype and as specific as single nucleotide polymorphisms (SNPs). The decision on which reference genome to use when several are available favors that most closely correlated at both general and specific layers with the organism under study.

Millions of years of divergent evolution may cause problems when comparing distantly related genomes. Even closely related species may share sequence homology, conserved synteny and genome architecture among only a small set of genes and regions after the effects of inversions, replications, translocations, insertions, deletions, duplications and a myriad number of other genetic mutations. Evolutionary pressure can also cause varying genome size and karyotype divergence.

Two genomes were available for use as reference sequence within the *Fusarium *plant pathogen complex, *Giberella zeae *(*F. graminearum*) [14] and *Nectria haematococca *(*F. solani *f.sp. *pisi*) [15]. The cereal crop fungal pathogen *Fusarium graminearum *had a nearly complete genome sequence [14], a physical map [16] had the same chromosome number and shared many gene expression patterns with the pathogenic *F. virguliforme *[17] (Yuan and Lightfoot, unpublished).

The objective was to cluster and align discontinuous BAC-end sequences from *F. virguliforme in silico*, using sequence resources available for *F. graminearum*. Three steps would be taken. First, a method for grouping thousands of discrete DNA fragments from an uncharacterized genome based on the reference genome was developed. Second, the reference genome was annotated for genic (predicted exon) content. Finally, the random BES matches to the reference genome were presented in a linear map format, based on proximity of sequence identities from both ends of each BAC. After these steps, a prediction of regions spanned by the BAC clones could be made and displayed in an informative manner.

## Methods

### Sequences used for genome comparison

In June, 2007, there were 4,152 *F*. *virguliforme *BES available on Genbank [8,9]. The BES encoded 2,976,557 bp of *Fusarium virguliforme *DNA, at an average of 717 bp per record and an overall GC content of 53.9%. The 36,093,143 bp of *Fusarium graminearum *reference genome is represented in 511 sequence fragments located within 19 large (>20 Kbp) and 24 small (<20 Kbp) scaffolds that correspond to 4 chromosomes [16] and have a GC content of 48.4%.

### Sequence processing

**Figure 1:**
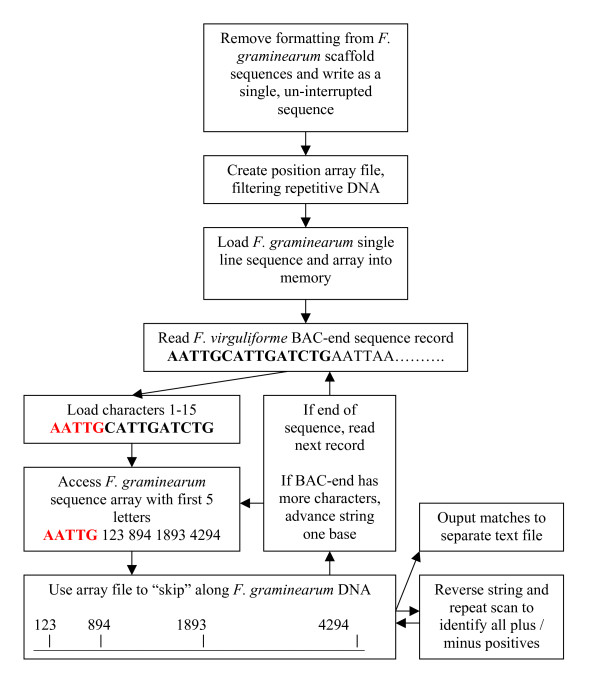
Program flow diagram for sequence comparison using 36 Mbp of *Fusarium graminearum *scaffold sequence as a reference for 4,152 *Fusarium virguliforme *BAC end sequences. All JAVA code used for this comparison is freely available from the authors.

The creation of an efficient comparison utility required a multi-program process (Figure 1). First, FASTA formatted *F. graminearum *sequence was processed to remove line feeds and headings. Second, the file was scanned for 7 bp sequences that contained all four bases (A, T, G and C). Motifs with all four bases were written to an array composed of 1,020 possible combinations of the first 5 base pairs (5 base repeats of A, T, G, or C were excluded from the analysis).

The array of sequence motifs and positions was loaded into a second program, followed by the nucleotide-only reference sequence. Fasta formatted BES was input in series, with each BES screened against the *F. graminearum *genomic sequence. In brief, a five base nucleotide sequence from the BES was used to access the array of sequences, allowing the program to attempt to align only those sequences that shared the first five base pairs of each 15 bp fragment. In addition, the reverse complement of each BES was also tested. Execution times for comparison of 4,152 BES against 36 Mbp of *F. graminearum *genomic DNA were less than three hours. Output from the comparison program was in the form of the reference genome sequence location, BES ID and matching sequence location. If the BES match was from the reverse complement of a sequence a "-" notation was made.

The identification of exonic fragments was carried out using TimeLogic^® ^GeneDetective™ (Active Motif Inc., Carlsbad, CA) In order to directly relate the exons to the associated BES fragments and to accommodate the processing restrictions of Gene Detective, the identical 36 Mbp single sequence used for sequence comparison was divided into smaller, 999 bp linear order fragments, then tested these against the non-redundant fungal EST database (downloaded from NCBI on Nov 16, 2006; 886,100 fungal ESTs). We used only the top two ranked hits for each sequence and removed overlapping results.

A Windows XP o/s, w/2 GHz processor and 512 MB RAM was used for analyses (other than Gene Detective) performed in this experiment. A map using the 19 large sequence *Fusarium graminearum *scaffolds as a framework was prepared in MapChart [18], with matching *F. virguliforme *clones and exon content of *F. graminearum*. Verification of alignments was performed using the BLAST2Sequence utility at NCBI [19].

## Results and discussion

### *Fusarium graminearum *scaffold-based map

To expand the use of reference genomes, sequence testing techniques must allow for variations in genetic drift and be capable of reporting associations with varying degrees of certainty [20-22]. In order to meet both of these demands, fifteen base pair “windows” were taken from BAC-end sequences and checked against the reference genome. In addition, each window was reversed and checked against the reference, allowing for change in read direction of BES or of the reference sequence. A fifteen base pair fragment represents a probability of random association every 1 Bbp (4^15^). Smaller base pair probes will result in an exponential increase in reported associations. For example, reducing the base pair window to 10 would result in a probability of random association every 1 Mbp (4^10^), with loss of accuracy as probability dictates the likelihood of a random match in the reference genome.

As Figure 1 indicates, five bp sequence arrays were used to position the *F. virguliforme *BAC-end probe sequence against the *F. graminearum *scaffold sequence.

These arrays performed two vital tasks. First, they increased processing speed by nearly 1000× over non-arrayed comparisons. The second advantage related to how the array was prepared. Each 5 bp sequence was written to the array only after the next two bases were added, then this 7 bp fragment was tested for the inclusion of all 4 nucleotides (A, T, G and C). This provided an initial screen of low complexity DNA. It was found that this complexity test was too stringent when tested on 5 bp fragments, and too lax at 8 bp.

Identifying conserved regions between *F. virguliforme *BACs and *F graminearum *DNA led to the detection of a total of 46,590 15 bp matches. These matches allowed the integration of *F. virguliforme *BAC-end sequence into the linear order scaffold map of *F graminearum*.

There were two criteria built into this experiment: First, a 15 bp match had to occur; Second, another 15 bp match *from the other end *of the clone was required within 200 Kbp on the reference sequence. Clones meeting these two criteria were separated into three groups, based on the expected bidirectional nature of BAC end sequence data from the same clone and the unidirectional nature of the *F. graminearum *scaffolds. The first group were those that had alternating match direction between the two BAC ends *and *were contained within the same scaffold subsequence. The second group was identical to group 1, except that the two ends span at least two scaffold subsequences. The third group were those that had unidirectional matches between the two BAC ends, regardless of scaffold subsequence position.

**Figure 2:**
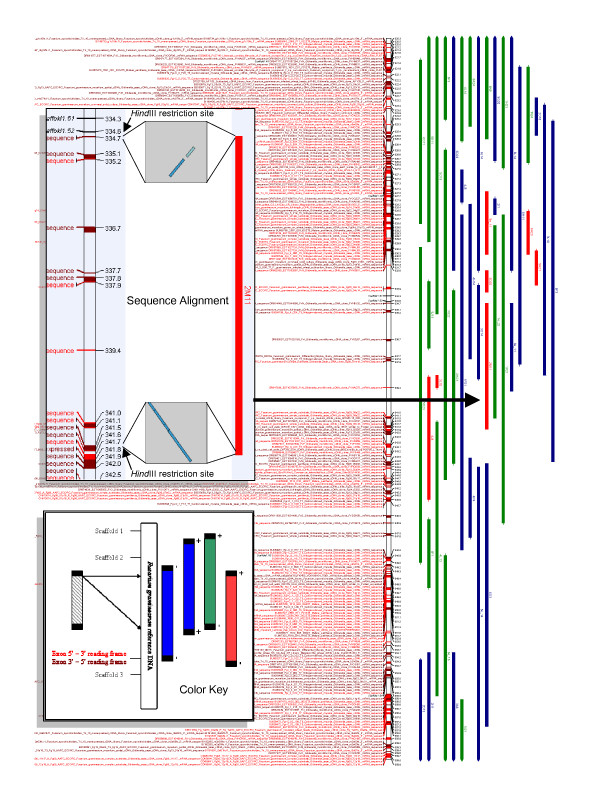
Graphical representation of predicted sequence alignments between *Fusarium graminearum *reference sequence and *Fusarium virguliforme *BAC-end sequences. Inset key panel shows how color of predicted match was determined. A match from *both ends *of the BAC within 200 kb was required for display. Uni-directional matches are indicated in blue. Reversed order matches are indicated in green if they cross a scaffold marker (indicating a discontinuous sequence) and red if they are entirely within a single, continuous scaffold. *Fusarium graminearum *exonic fragments are indicated in red (5'-3') and magenta (3'–5'). Inset sequence alignment panel illustrates the strength of the match between both ends of clone “2M11” and scaffold 1.52 (1e-^35 ^top and 3e-^150^, bottom) The presence of *Hind*III restriction fragments was verified in the *F. graminearum *sequence, as would be predicted by the position of 2M11.

A total of 2,560 putatively syntenic associations were identified, encompassing 247,012,452 bp of *F. graminearum *DNA (6.8× genome coverage). A total of 1,339 *F. virguliforme *clones were identified that exhibited plus *and *minus strands matching up to 200,000 bp apart. The total DNA covered by +/- alignments was 127,482,241 bp. These clones were separated into 531 that fit within a single scaffold subsequence (30,915,996 bp; red clones, Figure 2) and 808 that span more than one subsequence (96,566,245 bp; green clones, Figure 2) In addition, 1,221 clones exhibited plus and plus *or *minus and minus strands matching up to 200,000 bp apart are indicated on the map in blue. The total DNA covered by +/+ or -/- matches was 119,530,211 bp.

As an example, the match of clone 2M11 (gi #s 38262078 and 38262180) with *F. graminearum *scaffold 1.52 is shown in the sequence alignment panel of Figure 2. The 5' match was in two fragments, (181/230 bp, 1e-^35^; 95/119 bp, 8e-^15^) around bp position 3,347,394 (3.347 on the map). The 3' match was reversed, with 554/683 bp and an expect value of 3e-^150 ^around bp position 3,418,000 (3.418 on the map). Of additional interest was the location of perfectly preserved *Hind*III restriction sites, 92 bases *upstream *(3,347,302 bp) in the 5' *F. graminearum *sequence and another at the *final position *(3,418,308 bp) of the 3' match (Figure 2, sequence alignment panel). The strength of the sequence matches identified and the nearly perfect correlation with *Hind*III sites used for cloning the BAC library provide strong evidence of the effectiveness of this sequence correlation technique.

At least 22,614 unique exons encompassing a total of 6,888,863 bp were detected in the *F. graminearum *scaffold sequences. The match direction of these sequences is indicated with red (forward) or magenta (reverse) text in the map. A total of 94 maps (spanning ~400 kb each) were generated by this procedure and are available as a self-extracting file [see Additional file 1], which includes a searchable excel file. Each map is viewable as a completely scalable enhanced metafile using the Microsoft™ Picture and Fax Viewer program.

### Future work

This report is important because it describes the use of *F. graminearum *sequence as a reference to develop genome resources for *F. virguliforme*. The conserved synteny inferred will greatly accelerate the genomic analysis of *F. virguliforme*. The map may also accelerate the discovery of pathogenic gene clusters [16]. Parallels in gene expression underlying the pathogenic mechanisms of both *F. virguliforme *and *F. graminearum *may be identified [17]. Strategies to reduce yield loss to SDS [4] may also be developed. For example, because *F. virguliforme *is a member of the *Fusarium *plant disease complex which contains many related pathogens of all the worlds major crops [5,6], development of genome based fungicides and seed treatments for one may have broad utility. Since *F. virguliforme *is a hemibiotrophic plant pathogen with a wide host range [1,5], tests against many model plant species to identify new resistance genes are underway. Further, since the perfect stage is not known and all extant strains appear to be of a single mating type [7], these maps represent a significant genetic tool. Finally, the BAC libraries present opportunities for functional genomics, since the vector is a binary T-DNA capable of both plant and fungal transformation mediated by *Agrobacterium *strains [8,9].

## Conclusion

The main power of this method lay in its ability to align *diverged *sequences. Because the program uses relationships between 15 bp sequences, the reference genome can exhibit significant change from the probe sequences, yet still effectively group fragments of DNA. This work is unique in that discontinuous sequences were used for the analysis, information not readily apparent such as match direction are presented and the JAVA programs used are freely available. The 94 maps represent an excellent resource for continuing research on *Fusarium virguliforme*.

## Abbreviations

BAC, bacterial artificial chromosome; BES, bacterial artificial chromosome end sequence; bp, base pair; SDS, soybean sudden death syndrome; SNP, single nucleotide polymorphism.

## Authors' contributions

JS conceived of the study; SA and DL provided critical review, interpretation of results and funding (DL), LB performed geneic annotation of the *F. graminearum *sequences. All authors read and agreed to the final manuscript.

## Supplementary Material

Additional file 1 A self-extracting file containing 94 maps generated by short sequence comparisons between *Fusarium virguliforme *and scaffold sequences from *Fusarium graminearum*. Each map is viewable using the Microsoft™ Picture and Fax Viewer program. The extracted files include a searchable Microsoft™ Excel file. Click here for file
